# Rhizosphere soil bacterial communities and nitrogen cycling affected by deciduous and evergreen tree species

**DOI:** 10.1002/ece3.9103

**Published:** 2022-07-13

**Authors:** Jiantong Liu, Xinyu Wang, Lin Liu, Xuefeng Wu, Zhichao Xia, Qingxue Guo

**Affiliations:** ^1^ Department of Ecology, College of Life and Environmental Sciences Hangzhou Normal University Hangzhou China; ^2^ Institute of Grassland Science, Key Laboratory of Vegetation Ecology of the Ministry of Education, Jilin Songnen Grassland Ecosystem National Observation and Research Station Northeast Normal University Changchun China

**Keywords:** actinorhizal plants, co‐occurrence network, mixed plantations, photosynthetic carbon, plant–soil interactions

## Abstract

Deciduous and evergreen trees differ in their responses to drought and nitrogen (N) demand. Whether or not these functional types affect the role of the bacterial community in the N cycle during drought remains uncertain. Two deciduous tree species (*Alnus cremastogyne*, an N_2_‐fixing species, and *Liquidambar formosana*) and two evergreen trees (*Cunninghamia lanceolata* and *Pinus massoniana*) were used to assess factors in controlling rhizosphere soil bacterial community and N cycling functions. Photosynthetic rates and biomass production of plants, 16S rRNA sequencing and N‐cycling‐related genes of rhizosphere soil were measured. The relative abundance of the phyla Actinobacteria and Firmicutes was higher, and that of Proteobacteria, Acidobacteria, and Gemmatimondaetes was lower in rhizosphere soil of deciduous trees than that of evergreen. Beta‐diversity of bacterial community also significantly differed between the two types of trees. Deciduous trees showed significantly higher net photosynthetic rates and biomass production than evergreen species both at well water condition and short‐term drought. Root biomass was the most important factor in driving soil bacterial community and N‐cycling functions than total biomass and aboveground biomass. Furthermore, 44 bacteria genera with a decreasing response and 46 taxa showed an increased response along the root biomass gradient. Regarding N‐cycle‐related functional genes, copy numbers of ammonia‐oxidizing bacteria (AOB) and autotrophic ammonia‐oxidizing archaea (AOA), N_2_ fixation gene (*nifH*), and denitrification genes (*nirK*, *nirS*) were significantly higher in the soil of deciduous trees than in that of the evergreen. Structural equation models explained 50.2%, 47.6%, 48.6%, 49.4%, and 37.3% of the variability in copy numbers of *nifH*, AOB, AOA, *nirK*, and *nirS*, respectively, and revealed that root biomass had significant positive effects on copy numbers of all N‐cycle functional genes. In conclusion, root biomass played key roles in affecting bacterial community structure and soil N cycling. Our findings have important implications for our understanding of plants control over bacterial community and N‐cycling function in artificial forest ecosystems.

## Graphical abstract

Short‐time drought had less effect on bacterial communities than plant life habit. Root biomass is the main factor driving changes in soil bacterial communities. Highly diverse forests have high N cycling rates and stable bacterial communities. Bacterial taxa show different response along root biomass gradient.

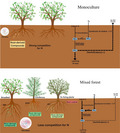

## 1 INTRODUCTION

Global climate change is likely to increase frequencies and severity of droughts, imposing soil water deficit stress on trees, which will markedly reduce productivity of plantations and natural forests (Gillespie et al., 2020; Pardos et al., 2021). Compared to monocultures, diverse plant communities are more likely to increase productivity and be more resistant and resilient to drought (Chen et al., 2021; Pardos et al., 2021). Increasing evidence suggests that the different drought‐resistant abilities have been deeply impacted by soil microbial communities (Castro et al., 2019; Osburn et al., 2021). Changes in the bacterial community in the face of drought decline soil nitrogen (N) availability through impacting soil N cycling process (Chen et al., 2021; Wang et al., 2021). However, greater microbial diversity in more diverse forests is thought to be beneficial to facilitate efficient nutrient cycling process thus potentially better sustaining forest functions and stability compared to monospecific forests (Gillespie et al., 2020; Naylor & Coleman‐Derr, 2018).

The N fixation, nitrification, and denitrification are key N cycling processes in soils, which are largely determined by specific microbial guilds and tightly connected with soil properties including water availability and pH (Bowen et al., 2020; McCulloch et al., 2020). Symbiotic N fixation by N_2_‐fixing plants constitutes the largest natural input of N into forest ecosystems (Ngom et al., 2016). Ammonia‐oxidizing bacteria (AOB) and autotrophic ammonia‐oxidizing archaea (AOA) have been primarily used to study nitrifying microbial communities (Guo et al., 2021; Trivedi et al., 2019). Genes (*nirK* and *nirS*) reflecting denitrifying microbial communities encode nitrite reductase to transform nitrite to nitric oxide (Bowen et al., 2020; Moreau et al., 2015). A lower soil water availability reduced N cycling by limiting extracellular enzymatic activities and impacting the N fixation, denitrifying or nitrifying microbial communities (Bowen et al., 2020; Castro et al., 2019; Guo et al., 2021).

In addition to effects of soil properties, plants can also strongly regulate soil microbial communities and microbially driven N‐cycling processes through litter or diverse root‐derived inputs of labile organic compounds (Henneron et al., 2020; Mushinski et al., 2020). The rhizosphere soil hosts diverse microbial communities that are crucial to promote plant nutrient acquisition and resistance/ tolerance to abiotic stressors (Moreau et al., 2015; Naylor & Coleman‐Derr, 2018). The N_2_‐fixing plants enhance N fixation ability by promoting photosynthesis and root‐derived carbon releasing to rhizosphere soil. Symbiotic N fixation is costly, and allocation of labile carbon (C) to root nodules of N_2_‐fixing plants can be greatly reduced due to decreased photosynthetic C fixation during drought (Minucci et al., 2019). Plants are proposed to control soil N cycling in the rhizosphere to sustain their nutrition and growth (Henneron et al., 2020). However, the impacts on microbial community and N‐cycling functions among plants with different N demand in response to drought need more research.

McCulloch et al. (2020) showed that drought reduced symbiotic N fixation by reducing nodule biomass and nitrogenase activity in eight N_2_‐fixing species. Root‐nodule symbiosis of actinorhizal plants and bacteria of the genus *Frankia* is an important pathway of new N input into forest ecosystems, in addition to symbioses of legumes and *Rhizobium* bacteria (Ngom et al., 2016; Tobita et al., 2015). The actinorhizal plants play crucial roles in replenishing soil nutrients of agroforestry and improving bacterial community in degraded land (Farías et al., 2009; Ngom et al., 2016). Among actinorhizal plants, species belonging to the genus *Alnus* are distributed worldwide and have been cultivated in monoculture for revegetating or rehabilitating disturbed habitats and commercial production (Tobita et al., 2015; Uri et al., 2014). Henneron et al. (2020) concluded that acquisitive species with higher photosynthesis and N uptake induced a stronger acceleration of N cycling than conservative species in rhizosphere soil. Deciduous and evergreen trees represent opposite extremes along “leaf economics spectrum,” which ranks from acquisitive and fast‐growing to conservative and slow‐growing traits (Wright et al., 2004). Deciduous trees with greater specific root length and specific leaf area show higher N uptake and photosynthetic capacity, growing faster than evergreen trees (Baldocchi et al., 2010; Cantarel et al., 2015; Wright et al., 2004). Growth of the deciduous species is more closely coupled to soil nutrient availability than evergreen species (Gray & Schlesinger, 1983). Higher N demand of deciduous trees may accelerate N cycling by imposing different selective pressures on soil bacterial community structures compared with evergreen species. Therefore, in the present study, we examined responses of soil bacterial community and functions associated with deciduous and evergreen trees to short‐term drought. We predicted higher number of N‐cycling‐related bacteria species in soil of deciduous trees due to higher N demands than evergreen trees. In addition, the crucial factors driving soil bacterial community and N‐cycling function of deciduous and evergreen trees under soil water deficit were elucidated.

## 2 MATERIAL AND METHODS

### 2.1 Experimental design

*Cunninghamia lanceolata*, *Pinus massoniana*, *Alnus cremastogyne*, and *Liquidambar formosana* are important tree species in monocultures and mixed plantations (Wang et al., 2009; Wu et al., 2021). The first two are coniferous evergreen trees, and the latter two are broadleaf deciduous species. Soils collected from a degraded land (0–20 cm depth) were homogenized after removing gravels, roots and plant litter. One‐year‐old *C. lanceolata*, *P. massoniana*, *A. cremastogyne* and *L. formosana* trees collected from a local nursery garden near the experiment site (30°19 N, 120°23 E) and 30 individuals per species were planted in plastic pots (30 cm external diameter and 21 cm height, one individual per pot) in mid‐December 2018. After 5 months, we selected 16 individuals of similar size and growth performance per species (May 28, 2019). We subjected all species to two treatments (water vs. drought); soil water content was maintained at approximately 80% field capacity (approximately 27.8% soil water) in the well water treatment and 30% field capacity (approximately 10.6% soil water) in the drought treatment. The pots were weighed every day to monitor soil water content and the lost water was replenished in time. Eight replicates per treatment were used. The experiment was conducted in a greenhouse at Hangzhou Normal University, China (30°19 N, 120°23 E).

### 2.2 Gas exchange measurements and harvesting

The net photosynthetic rate per treatment (eight replicates) was measured using an LI‐6400 photosynthesis system (Li‐Cor) under the following conditions: photosynthetic photon flux density 1500 μmol m^−2^ s^−1^, 28°C leaf temperature, 70% relative humidity, and 400 μmol mol^−1^ CO_2_. Measurements were made from July 29 to August 1, 2019. We harvested all plants on August 30, 2019, and all samples (root, stem, and leaf) were dried at 75°C for 72 h.

### 2.3 Rhizosphere soil physicochemical properties

Soil adhering to root was defined as rhizosphere soil. Rhizosphere soil samples were collected by carefully removing soil from the root surface after harvesting the plants. Four soil replicates of *P. massoniana* in the water treatment were discarded because of technical errors during transport. Subsamples of rhizosphere soil were air‐dried to measure pH, total phosphorus, available phosphorus, total N, and ammonium (${\mathrm{NH}}_{4}^{+}$‐N) and were determined according to Guo et al. (2019). In brief, soil‐water suspension (1:2.5 w/v) was used for pH determination. Soil extractions with 50 ml 2 M KCl were used to determine ${\mathrm{NH}}_{4}^{+}$. Soil digested by H_2_SO_4_ and HClO_4_ was used to measure total phosphorus through molybdenum blue colorimetry. Soil extractions with sodium bicarbonate was used to determine available phosphorus. The soil organic matter was measured according to Guo et al. (2021). Summary of soil properties is shown in Table S1. Further subsamples of fresh rhizosphere soil were stored at 4°C, and activities of β‐1,4‐N‐acetylglucosaminidase (NAG) and β‐D‐glucosidase were assessed using respective ELISA kits. NAG and β‐D‐glucosidase are related to chitin and cellulose degradation, respectively (Jing et al., 2020).

### 2.4 16S rRNA sequencing and real‐time quantitative PCR

Soil subsamples were stored at −80°C until DNA extraction. Total DNA was extracted from fresh soil (0.5 g) using a kit (Omega Bio‐tek), and DNA quality was assessed using 1% agarose gel electrophoresis. The 16S primers 338F (5′‐ACTCCTACGGGAGGCAGCAG‐3′) and 806R (5′‐GGACTACHVGGGTWTCTAAT‐3′) targeting the V3–V4 region were used for PCR amplification, followed by paired‐end sequencing on a MiSeq platform (Illumina; Guo et al., 2021). The PCR amplification steps followed: initial denaturation (3 min) at 95°C, 27 cycles (30 s) at 95°C, annealing (30 s) at 55°C, elongation (45 s) at 72°C, and a final extension (10 min) at 72°C. PCR reactions were performed in triplicate 20 μl mixture. Raw FASTQ reads were demultiplexed, quality filtered. More details concerning the process on raw reads are shown in the supplementary materials. All bacterial sequences were assigned to operational taxonomic units (OTUs) using the SILVA database and the UPARSE pipeline at 97% similarity (Quast et al., 2013).

DNA extracts were also subjected to quantitative PCRs (qPCRs) to quantitate copy numbers of N‐cycling genes: ammonia monooxygenase (amoA) genes of AOA, AOB, *nifH*, *nirS* and *nirK*. The following primer combinations were used: AOA forward: 5′‐ATGGTCTGGCTWAGACG‐3′, reverse: 5′‐GCCATCCATCTGTATGTCCA‐3′; AOB forward: 5′‐GGAGRAAAGCAGGGGATCG‐3′, reverse: 5′‐CTAGCYTTGTAGTTTCAAACGC‐3′; *nifH* forward: 5′‐TGCGAYCCSAARGCBGACTC‐3′, reverse: 5′‐ATSGCCATCATYTCRCCGGA‐3′; *nirS* forward: 5′‐AACGYSAAGGARACSGG‐3′, reverse: 5′‐GASTTCGGRTGSGTCTTSAYGAA‐3′; and *nirK* forward: 5′‐TGCACATCGCCAACGGNATGTWYGG‐3′, reverse: 5′‐TGCACATCGCCAACGGNATGTWYGG‐3′. More details concerning the process on qPCR are shown in the supplementary materials. Standard curves for each functional gene were produced according to Frey and Rieder (2013).

### 2.5 Statistical analyses

We used a two‐way analysis of variance (ANOVA) to identify the effects of species, drought, and their interaction terms on plant biomass, enzyme activity, and bacterial alpha‐diversity. We named a factor ‘leaf habit’ to indicate deciduous and evergreen tree species in the present study in order to compare their effects combined with drought on net photosynthetic rate, enzyme activity, qPCR results, and bacterial alpha‐diversity using two‐way ANOVA following Tukey's *b* post hoc test in case of significant interactions. Relative abundances of bacterial were analyzed at phylum and genus level between deciduous and evergreen trees with independent‐samples *T* test. Bacterial beta‐diversity was ordinated using nonmetric multidimensional scaling (NMDS) based on Bray–Curtis distances. PERMANOVA using the “adonis” function of the “vegan” in R software was used to find significant differences in soil bacterial community dissimilarity (Oksanen et al., 2020). “Ward. D2” function was used to perform hierarchical clustering analysis based on Bray–Curtis distances of bacterial OTUs from all samples, and the silhouette width method in R decided the number of clusters to be used (Xiong et al., 2021). The Spearman correlations between characters of bacterial community (the relative abundance of the major bacterial phyla), soil properties and plant growth versus functions in enzyme activities and N‐cycling‐related genes. The multiple regression model with variance decomposition analysis was applied to estimate the importance of the importance of soil, plant, and bacterial characteristics in driving the studied enzyme activities and N‐cycling‐related functional genes following descriptions in Jiao et al. (2020). After checking potential collinearity, distance‐based redundancy analysis (db‐RDA) was also performed using ‘vegan’ package. Network analysis was conducted and visualized using Cytoscape v. 3.5 and Gephi, respectively (Bastian et al., 2009; Shannon et al., 2003). Robust Spearman correlation scores (Spearman's |*r|* > .5) and statistically significant correlations were tested (*p* < .01). Network complexity and hub nodes in co‐occurrence networks were tested as described by Xiong et al. (2021). Soil bacterial community change along the root biomass gradient was analyzed using threshold indicator taxa analysis (Baker et al., 2015). We used bootstrapping (*n* = 500) to estimate uncertainty around the environmental change points for each indicator taxa. Structural equation models (SEM) were used to test the effects of root biomass on each N‐cycling function using the ‘lavaan’ package (Rosseel, 2012). The model was fitted according to chi‐squared tests, a high comparative fit index (≥0.90), a low root means square error of approximation index (≤0.1), and a low standardized root means square residual index (≤0.1).

## 3 RESULTS

### 3.1 Plant performance in carbon fixation and biomass production

The largest root biomass was produced by *A. cremastogyne* in the water treatment (Table 1). Root biomass of *L. formosana* and *A. cremastogyne* was significantly smaller in the drought than in the water treatment, but root biomass of *C. lanceolata* and *P. massoniana* was not significantly smaller in the drought than in the water treatment (Table 1). Deciduous trees showed significantly higher net photosynthetic rates than evergreen both at well water (12.86 vs. 7.46 μmol m^−2^ s^−1^) and drought treatment (5.45 vs. 1.63 μmol m^−2^ s^−1^; Figure S1).

**TABLE 1:** Growth characteristics of four tree species in response to two soil water levels

	Aboveground biomass (g)	Root biomass (g)	Total biomass (g)
*Liquidambar formosana*			
Control	59.09 ± 1.77a	6.86 ± 0.38c	65.95 ± 1.64a
Drought	30.95 ± 1.92b	5.22 ± 0.29d	36.17 ± 1.97b
*Alnus cremastogyne*			
Control	23.70 ± 1.54c	12.66 ± 0.46a	36.35 ± 1.80b
Drought	14.33 ± 1.20de	8.39 ± 0.35b	22.72 ± 1.42c
*Cunninghamia lanceolata*			
Control	19.60 ± 1.64 cd	4.50 ± 0.29de	24.10 ± 1.87c
Drought	12.13 ± 1.47e	3.02 ± 0.29e	15.15 ± 1.70d
*Pinus massoniana*			
Control	4.32 ± 0.55f	0.56 ± 0.05f	4.87 ± 0.54e
Drought	4.04 ± 0.69f	0.49 ± 0.07f	4.53 ± 0.68e
Significant effects			
Species	(243.616)***	(303.968)***	(255.620)***
Drought	(105.581)***	(62.706)***	(122.493)***
Species × drought	(29.325)***	(13.596)***	(26.346)***

*Note*: A two‐way analysis of variance was used to test the effect of plant species, drought, and their interactions on growth characteristics. Different lowercase letters in the column indicate significant differences. Significant effects of factors and interactions (×) are indicated. Numbers in brackets indicate *F* values.

Abbreviation: NS, not significant.

*** *p* ≤ .001.

### 3.2 Composition, diversity, and structure of bacterial community

**FIGURE 1:**
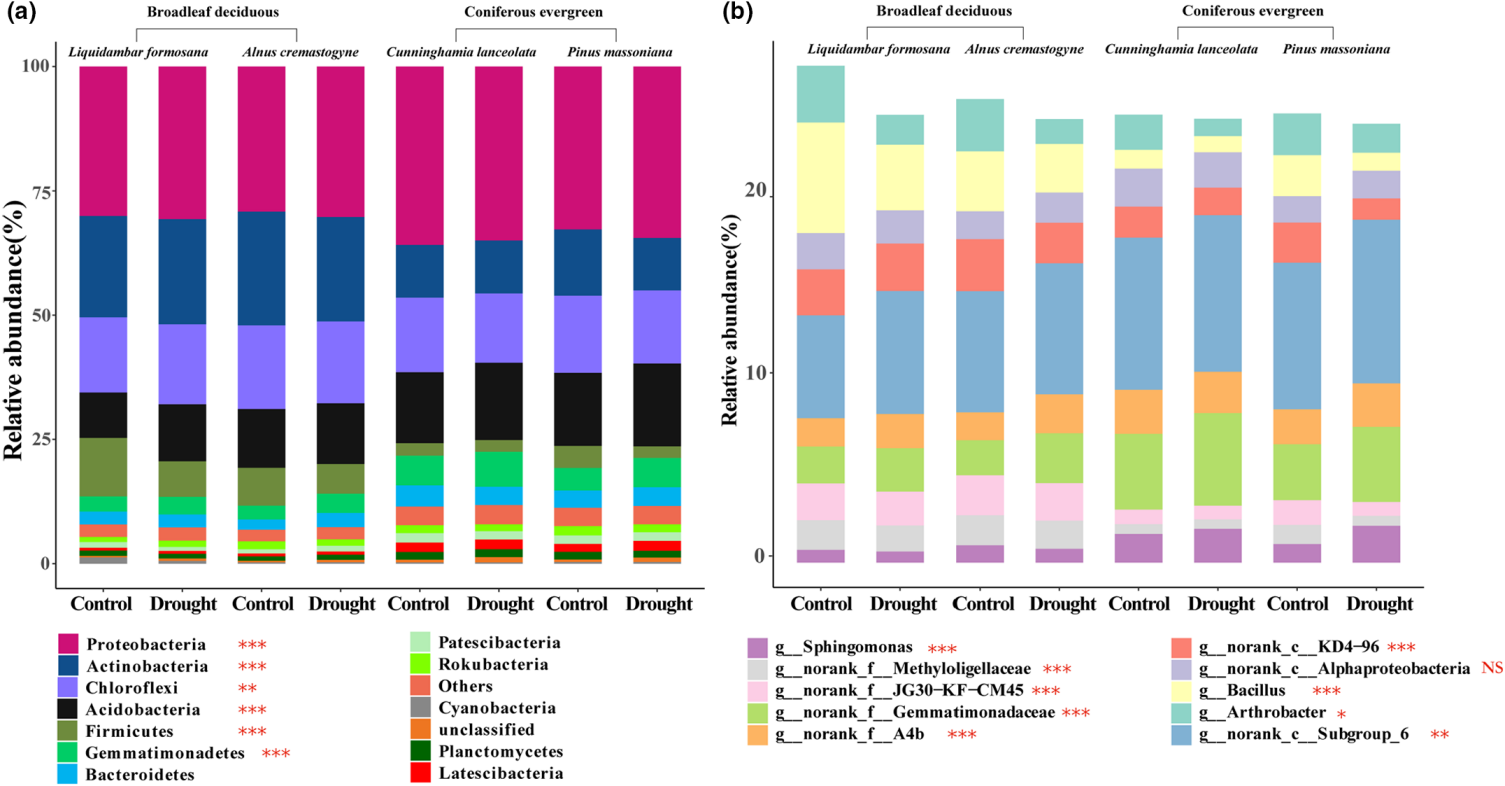
Relative abundances in the soil of four tree species exposed to two water regimes at the phylum (a) and genus level (b), respectively. Only the top 10 genera are shown. Deciduous tree species (*Alnus cremastogyne*, an N_2_‐fixing species, and *Liquidambar formosana*) and two evergreen trees (*Cunninghamia lanceolata* and *Pinus massoniana*). Independent samples *T* test was taken to compare differences between deciduous and evergreen species. NS: *p* > .05, *: .05 *≤ p* < .01, **: .01 *≤ p* < .001, ***: *p* ≤ .001

Proteobacteria, Actinobacteria, Chloroflexi, Acidobacteria, Firmicutes, and Gemmatimondaetes were the six most abundant soil bacterial phyla. The drought treatment did not significantly affect the relative abundances of each phylum (Figure 1a). However, Actinobacteria and Firmicutes in the soil of deciduous trees showed higher relative abundances, whereas those of Proteobacteria, Acidobacteria, and Gemmatimondaetes were lower in deciduous than in coniferous tree soils (Figure 1a). The dominant bacterial taxa at genus level were also significantly different between deciduous and coniferous tree soils (Figure 1b). For example, the relative abundance of *Arthrobacter* and *Bacillus* in deciduous tree soils were significantly higher than in coniferous.

**FIGURE 2:**
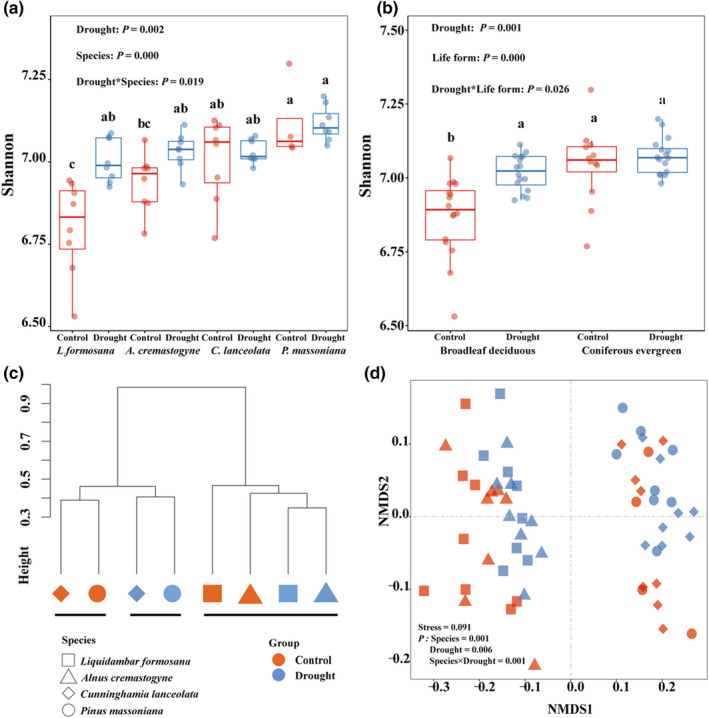
Bacterial alpha‐ and beta‐diversity in the soil of two deciduous (*Alnus cremastogyne* and *Liquidambar formosana*) and two evergreen (*Cunninghamia lanceolata* and *Pinus massoniana*) tree species in response to two water regimes. (a) Bacterial Shannon diversity of each species at operational taxonomic unit level, (b) Bacterial Shannon diversity of both deciduous and evergreen species combined. A two‐way analysis of variance was used. Post hoc tests were used to test differences among treatments with Tukey's *b* tests. Different letters indicate significant differences. (c) Hierarchical clustering analysis and (d) nonmetric multidimensional scaling (NMDS) based on Bray–Curtis distances

Shannon diversity in the *L. formosana* soil community after the drought was significantly higher than under well‐watered conditions (Figure 2a). Shannon diversity of *C. lanceolata* and *P. massoniana* soil communities tended to be higher than those of communities associated with *L. formosana* and *A. cremastogyne*. Soil bacterial alpha‐diversity in the soil of deciduous species was significantly increased by drought, whereas that in the soil of coniferous species did not differ significantly between water and drought treatments (Figure 2b). Plants significantly affected richness of bacterial community (Figure S2). Regarding bacterial beta‐diversity, clustering and NMDS analyses showed a clear separation between broadleaf deciduous and coniferous trees soil communities (Figure 2c,d).

**FIGURE 3:**
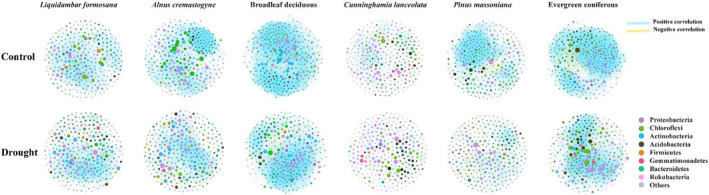
Co‐occurrence network of the four tree species and two plant leaf habits in the control (water) and drought treatments. *Cunninghamia lanceolata* and *Pinus massoniana* belong to the evergreen species, *Alnus cremastogyne* and *Liquidambar formosana* belong to the deciduous species

Drought and species exerted specific effects on network traits, such as clustering coefficients, positive/negative relationships, modularity, and hub node number (Figure 3; Figure S2; Table 2). Regarding plant leaf habit, drought declined positive relationships, average degree, clustering coefficients, and hub nodes but increased negative relationships and path distance. Modularity was reduced in broadleaf deciduous trees but increased in coniferous evergreen trees in the drought treatment (Table 2; Figure S3).

**TABLE 2:** Traits of bacterial co‐occurrence networks of four tree species and two plant leaf habits in response to control (water) and drought treatments

	Treatment	Positive edge	Negative edge	Average degree	Modularity	Average clustering coefficient	Average path distance	Hub node
Leaf habit								
Broadleaf deciduous	Control	7547	20	51.128	0.440	0.592	2.146	123
	Drought	5726	72	39.044	0.348	0.525	2.408	77
Coniferous evergreen	Control	8304	119	56.341	0.343	0.607	2.204	129
	Drought	4502	150	31.327	0.416	0.482	2.631	47
Species								
*Liquidambar formosana*	Control	2283	14	15.896	0.512	0.472	3.444	0
	Drought	2438	107	17.196	0.434	0.496	3.668	1
*Alnus cremastogyne*		4002	1	27.311	0.492	0.586	3.123	50
	Drought	1706	65	12.621	0.589	0.479	3.888	0
*Cunninghamia lanceolata*	Control	2017	105	14.937	0.558	0.478	3.487	0
	Drought	1388	68	10.189	0.634	0.431	4.065	0
*Pinus massoniana*	Control	4273	83	30.455	0.370	0.561	3.124	56
	Drought	1146	178	9.355	0.614	0.416	4.391	0

### 3.3 Nitrogen‐cycling functions

**FIGURE 4:**
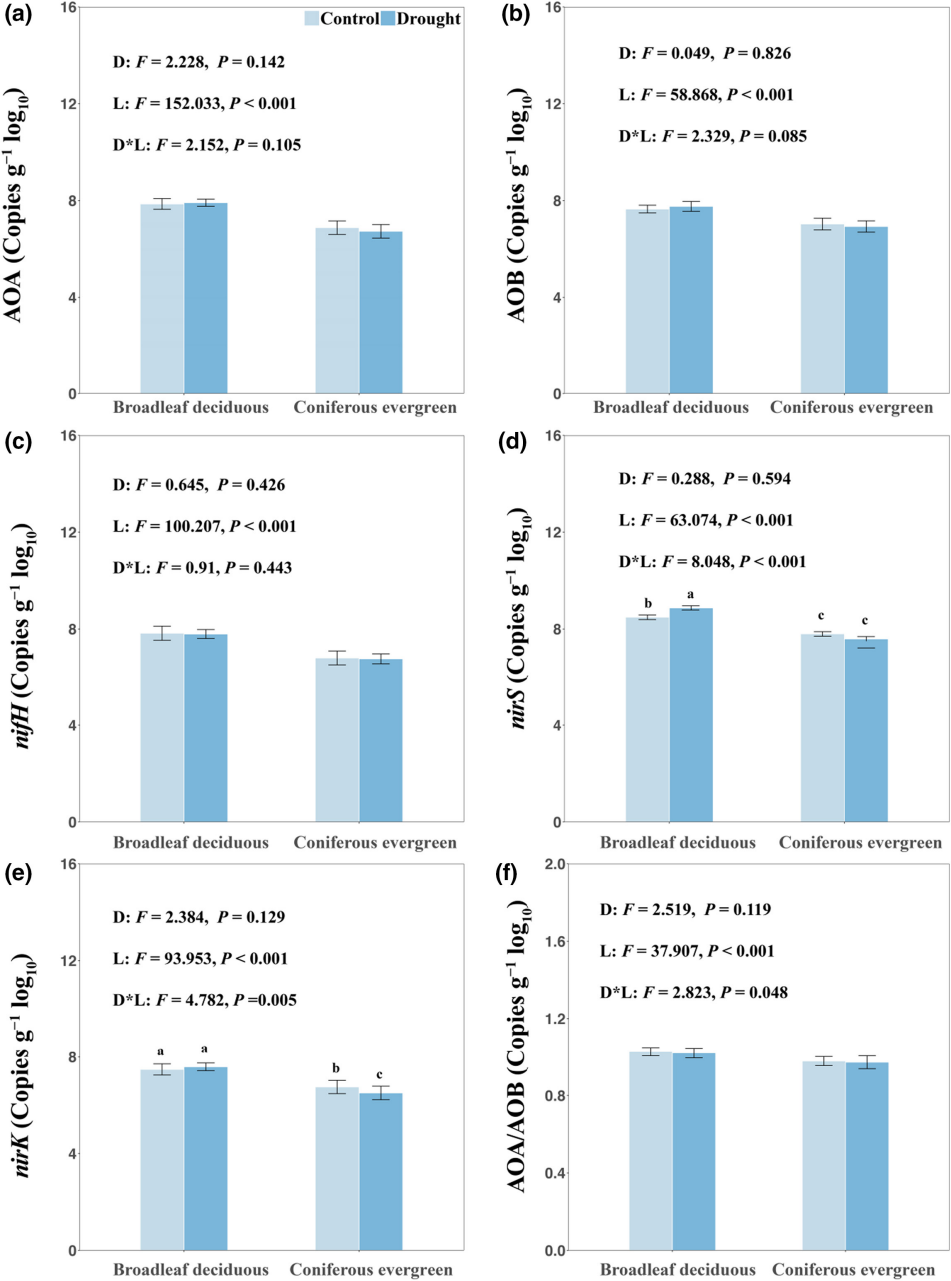
Copy numbers of N‐cycling‐related autotrophic ammonia‐oxidizing archaea (AOA) (a), ammonia‐oxidizing bacteria (AOB) (b), *nifH* (c), *nirS* (d), and *nirK* (e). The ratio between AOA and AOB was shown (f). A two‐way analysis of variance was used to test the effects of plant leaf habit, drought, and their interactions on these genes. *p‐*values of each factor are shown. Post hoc tests were used to test differences among treatments with Tukey's *b* tests. Different letters indicate significant differences. *Cunninghamia lanceolata* and *Pinus massoniana* belong to the evergreen species, *Alnus cremastogyne* and *Liquidambar formosana* belong to the deciduous species

Soil β‐D‐glucosidase in *P. massoniana* and NAG in *C. lanceolata* showed the highest activity in the water treatment (Figure S4a,b), and NAG activity was significantly higher in the soil of evergreen than in that of deciduous trees (Figure S4). Soil AOA, AOB, *nifH*, and *nirS* copy numbers were significantly higher in the soil of deciduous trees than in evergreen (Figure 4). The ratio of AOA to AOB was also higher in the soil of deciduous trees (Figure 4f). These functional genes were less affected by drought than by plant leaf habit, and significant interaction effects of the two factors were found on the denitrification‐related genes *nirS* and *nirK*. Copy numbers of *nirS* in the soil of deciduous species were significantly higher in the drought than in the water treatment (Figure 4d), whereas in evergreen, *nirK* copy numbers were significantly lower in the drought than in the water treatment (Figure 4e).

### 3.4 Effects of root on bacterial community and N‐cycling functions

**FIGURE 5:**
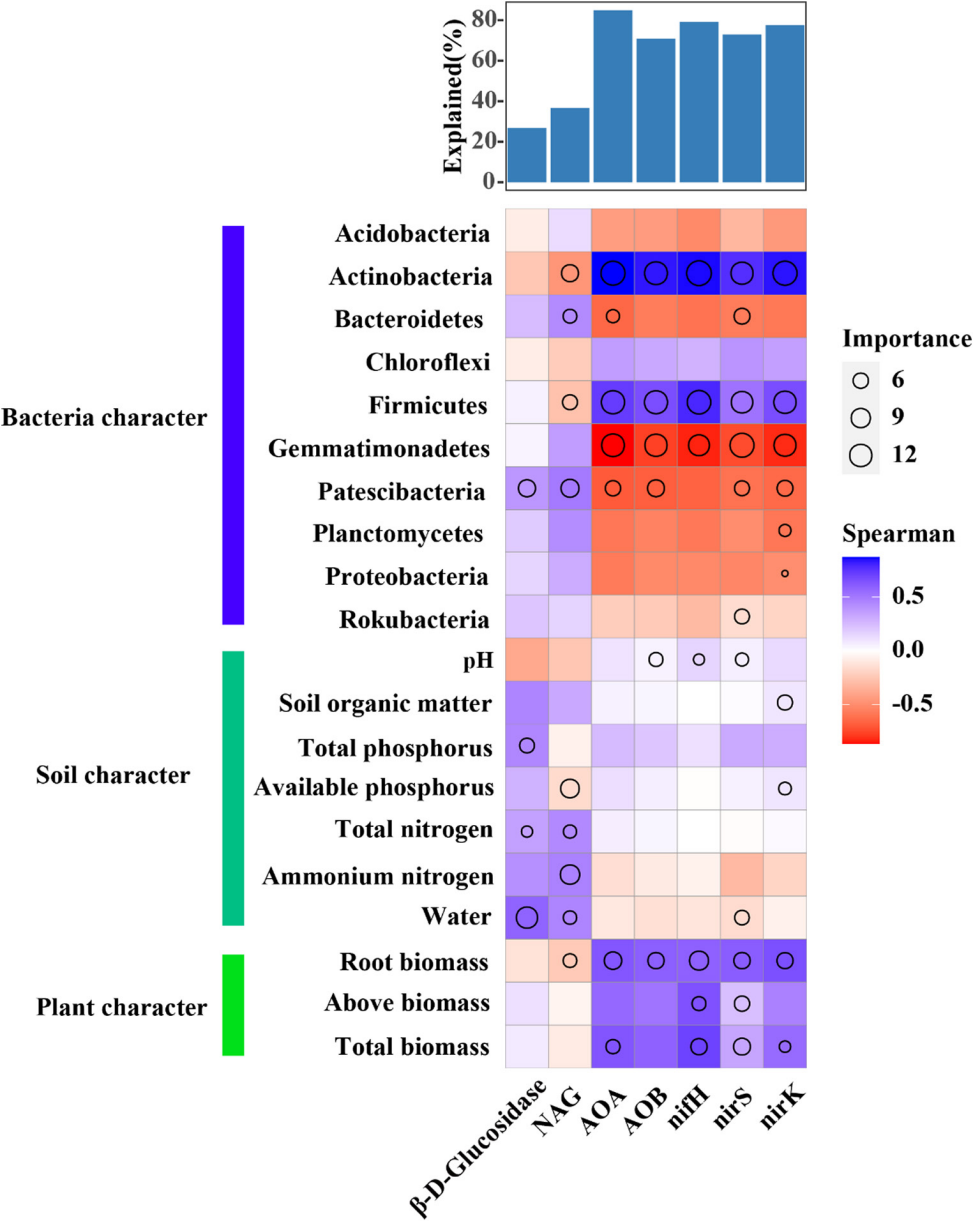
Potential biological contributions of plant, soil, and bacterial characteristics in enzyme activities and N‐cycling genes identified using correlation and random forest model. The Spearman correlations calculated between differences in plant, soil, and bacterial characteristics versus differences in enzyme activities and N‐cycling genes. The multiple regression model with variance decomposition analysis was applied. Circle size represents the importance of soil and plant characteristics in driving major bacterial taxa. Colors represent Spearman correlations. AOA, ammonia‐oxidizing archaea; AOB, ammonia‐oxidizing bacteria; NAG, β‐1,4‐N‐acetylglucosaminidase

Root biomass was a key factor in predicting diverse functions of soil microbial community (Figure 5). NAG activity, copy numbers of *nifH*, AOB, AOA, *nirK* and *nirS* was positively related with root biomass, and abundance of Actinobacteria and Firmicutes, while negatively related with the abundance of Gemmatimonadetes (Figure 5). Root biomass and soil properties explained 89.77% of the variation in bacterial communities, and RDA axis 1 explained 79.92%, with root biomass as the main factor affecting the variation of this axis (Figure S5).

**FIGURE 6:**
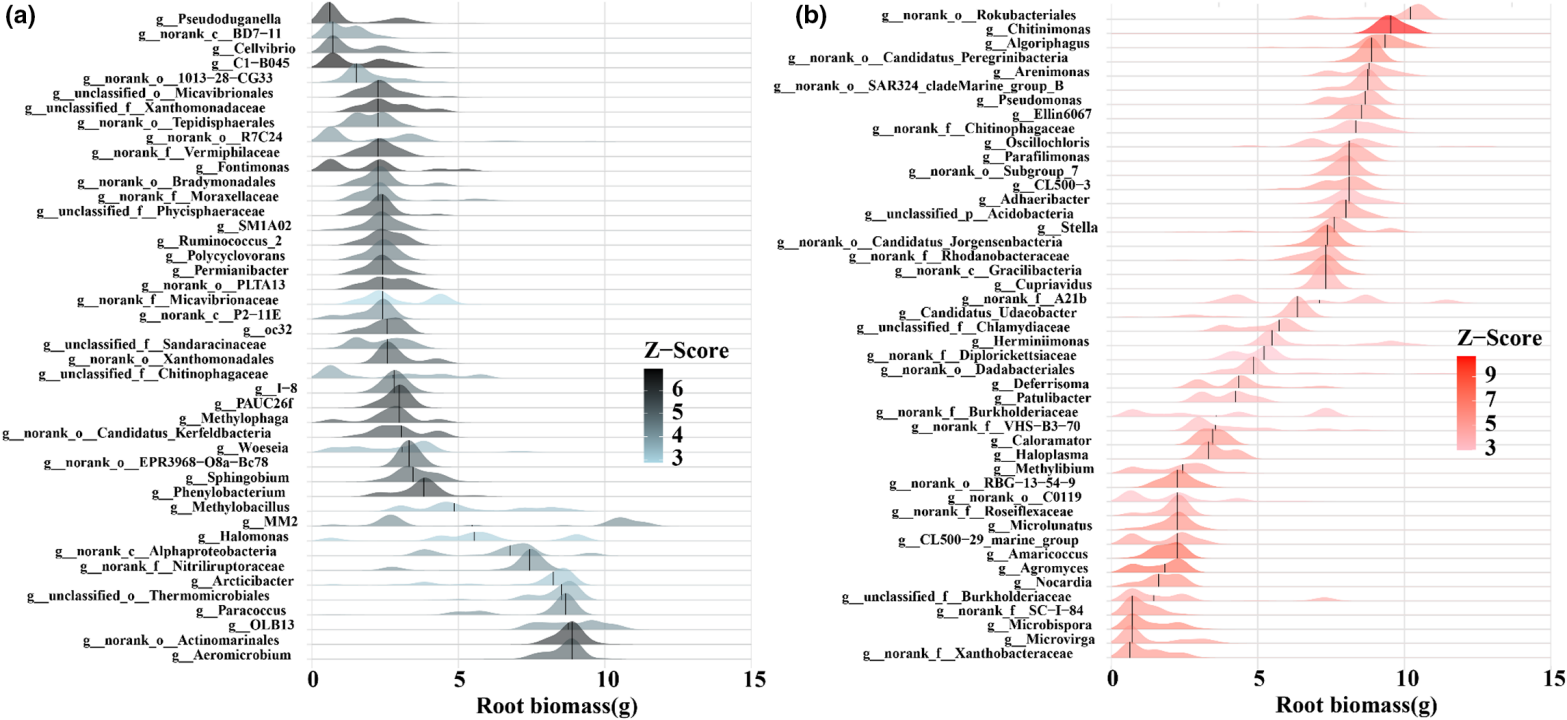
Threshold Indicator Taxa Analysis of soil bacterial community at genus level in responding to a root biomass gradient. Only taxa with pure (purity ≥0.95) and reliable (reliability ≥0.95) responses are plotted. Bootstrapping‐based (*n* = 500) probability density functions of negative responding bacteria taxa (blue) (a) and positively responding bacteria taxa (red) (b) along the root biomass gradient

Threshold indicator taxa analysis (TITAN) identified 44 bacteria genera with a decreasing response along the root biomass gradient and 46 taxa showed an increasing response (Figure 6). The negative responding bacteria taxa declined sharply at small root biomass (2.265 g), while the positive responding bacteria taxa increased at high root biomass (8.885 g; Figure S6). The most sensitive negative responding bacteria taxon (lowest change point value) was *Pseudoduganella* while the most sensitive positive responding bacteria taxon (highest change point value) was *Chitinimonas* (Figure 6).

**FIGURE 7:**
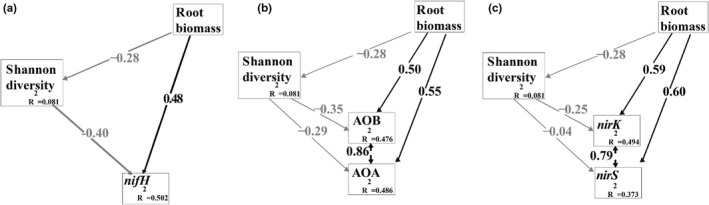
Structural equation models to assess relationships and effects of root biomass on soil alpha‐diversity and N‐cycling‐related genes (a‐c). AOA, ammonia‐oxidizing archaea; AOB, ammonia‐oxidizing bacteria

The SEM explained 50.2%, 47.6%, 48.6%, 49.4%, and 37.3% of the variability in copy numbers of *nifH*, AOB, AOA, *nirK*, and *nirS*, respectively (Figure 7). Root biomass negatively affected bacterial Shannon diversity, but the effect was not statistically significant. However, root biomass exerted significant positive effects on copy numbers of all N‐cycle functional genes (Figure 7); the correlation coefficients of *nifH*, AOB, AOA, *nirK*, and *nirS* were 0.48, 0.50, 0.55, 0.59, and 0.60, respectively.

## 4 DISCUSSION

Soil microbiomes could play essential roles in forest ecosystem responses to increasing anthropogenic global climate changes. We tested the individual and interactive effects of plant leaf habit and drought on soil bacterial community structure and N cycling function. Our results showed that bacterial community and N cycling function were interactively affected by plant leaf habit and drought.

### 4.1 Effects of plant and drought on bacterial community

The drought‐induced changes in diversity and taxonomic composition of soil bacterial community depend on water availability, soil physiochemical properties and plant physiological responses (Bastida et al., 2017; Han et al., 2021; Naylor & Coleman‐Derr, 2018). Drought significantly reduced SOM, TN, and ${\mathrm{NH}}_{4}^{+}$ of rhizosphere soils (Table S1) and induced changes in the abundance, diversity, and network structure of the bacterial community in this study (Figures 1, 2, 3; Figure S2). Nutrient competition among microbes and osmotic stress affects bacteria species (Chodak et al., 2015; Xie et al., 2021). The bacterial genera, for example, *Arthrobacter* and *Bacillus*, were reduced by drought, suggesting drought‐sensitive species belonging to the two genera were strongly inhibited by the declining water availability (Chodak et al., 2015; Xie et al., 2021). Plants also impose strong selective pressure on the bacterial community by recruiting species‐specific microbes in response to water deficit (Ehlers et al., 2020; Guo et al., 2019, 2021). In this study, co‐occurrence network results revealed a better connected and more stable bacterial network of rhizosphere soil surrounding *L. formosana* compared to *A. cremastogyne* with a greater decline in positive relationships, average degrees, and clustering coefficients but an increase in average path lengths after drought (Figure 3). Mutualistic relationships in the plant–soil continuum have been reported between bacterial communities and plants considering resistance to drought (Naylor & Coleman‐Derr, 2018). The more stable and connected network of bacterial community can be less affected by drought (Jiao et al., 2020).

### 4.2 Crucial role of root biomass in shaping soil bacterial community and function

Our results provided convincing evidences that the plant leaf habit imposed stronger impacts on bacterial community than short‐term drought. Greater variations happened in the relative abundance of dominant bacteria, richness and diversity (alpha‐ and beta), and N‐cycling function between deciduous and evergreen plants than drought (Figures 1, 2, and 4). Plant leaf habit had no significant effects on soil physiochemical traits (Table S1), which demonstrated an important role of plant characteristics in shaping the divergent bacterial community. The two deciduous species recruited higher relative abundance of plant growth‐promoting bacteria genera *Arthrobacter* and *Bacillus* than the two evergreen species (Figure 1). The random forest analysis and db‐RDA results confirmed the important role of plant characteristics, particularly root biomass, in driving bacterial structure and functions (Figure 5; Figure S5). Further analysis identified by TITAN showed that different indicators of bacterial taxa at the genus level along the smaller to larger root biomass gradient from evergreen to deciduous species (Figure 6; Figure S6). The hub nodes from the co‐occurrence network were considered as potential keystone taxa in microbial community (Jiao et al., 2020). The changing bacterial taxa indicators affected by the root mass were probably the main reasons in driving changes in keystone taxa, connection, and modularity of the network (Figure 3; Figure S3).

We found that root biomass was a crucial factor in determining the composition and structure of the soil bacterial community. Effects of deciduous and evergreen trees on soil bacterial communities can be attributed to differences in plant photosynthetic carbon supply as well as to the varying amounts of carbon released to the soil through root exudates (Guo et al., 2019, 2021; Keller et al., 2021; Zhalnina et al., 2018). Analogous to the leaf economic spectrum aboveground (Donovan et al., 2011; Wright et al., 2004), the root of deciduous and coniferous species ranges from more competitive and fast‐growing traits to more conservative slow‐growing traits (Sun et al., 2021). Deciduous trees with higher growth rates and biomass production show higher root exudation rates and release more root‐derived carbon to the rhizosphere compared to evergreen trees (Emmett et al., 2020; Sun et al., 2021; Wang et al., 2021). Our results were consistent with previous studies showing that deciduous trees typically have a higher net photosynthetic rate than evergreen trees (Baldocchi et al., 2010; di Francescantonio et al., 2020; Wang et al., 2021), which implies a higher quantity of root‐derived carbon released into rhizosphere soil.

### 4.3 Deciduous trees accelerate soil N cycle

We hypothesized that deciduous trees would elicit N cycling more strongly by promoting N‐cycling‐related bacteria, compared to evergreen; significantly higher copy numbers of N‐cycle‐related genes in the soil of deciduous species compared to that of evergreen supported this hypothesis. Plant–soil microbe interactions in the rhizosphere are a predominant link in N cycling and supply (Moreau et al., 2019). Plants seem to be able to control N transformations mediated by diverse soil microbes in and near the rhizosphere (Bardgett et al., 2014; Galland et al., 2019; Moreau et al., 2019; Mushinski et al., 2020). Random forest analysis identified root biomass as having a key role in affecting N‐cycling functions (Figure 5). All studied N‐cycling genes were positively correlated with the relative abundance of Actinobacteria and Firmicutes but negatively correlated with Gemmatimonadetes (Figure 5). The SEM also demonstrated strong positive effects of root biomass on N‐cycling genes (Figure 7). Higher N demand of deciduous trees requires more N input to meet plant biomass accumulation by increasing the abundance of *nifH*. Soil nitrate (${\mathrm{NO}}_{3}^{-}$) is the major plant‐available form of N, and higher AOB and AOA levels accelerate nitrification. However, the denitrification‐related bacteria showed higher gene copies. Cantarel et al. (2015) observed that denitrification depends on the relative growth rate of plants. Higher *nirK* and *nirS* in the rhizosphere of deciduous species can stimulate transformation from ${\mathrm{NO}}_{3}^{-}$ to nitrous oxide and increase N loss, which may imply that bacteria outcompete plants regarding the use of ${\mathrm{NO}}_{3}^{-}$ resources (Moreau et al., 2015). In addition, denitrification inhibitors or stimulators through the release of organic compounds have been reported (Bardon et al., 2016; Moreau et al., 2019). However, the exact mechanisms underlying the simulation of denitrification in the plant rhizosphere remain to be elucidated.

The four tree species studied in this study are widely used to construct establish artificial plantations (Wang et al., 2009; Wu et al., 2021). Our results have important implications for our understanding of plants control over bacterial community and N‐cycling function in artificial forest ecosystems. A recent study across nine European countries assessed responses of mixed and monoculture forests to drought events between 1975 and 2015 (Pardos et al., 2021), which showed that drought resistance of mixed conifer‐deciduous broadleaf forests exceeded that of mixed broadleaf forests; this suggests an important role of plant leaf habit diversity in mixed forests (Figure S7). Our simple framework showed that different tree species with different roots (biomass, architecture, or exudates) recruited more diverse microbes to influence forest productivity (Figure S7). The diversity of mycorrhizal fungi associating with roots of different plant leaf habits contributes to niche separation with respect to acquisition and transport of soil N and water, which suggests reduced competition for these resources due to symbiotic mycorrhiza. Roots of N_2_‐fixing plants colonized by mycorrhiza show increased N fixation and drought resistance (Liu et al., 2020), and N_2_‐fixing plants increase soil N availability and promote the growth of adjacent plants (Chen et al., 2021; Minucci et al., 2019; Tang et al., 2019). Mixed forests greatly increase soil N availability compared to monocultures, by promoting N mineralization, increasing soil N retention, or reducing denitrification (Chen et al., 2021; Wang et al., 2021).

## 5 CONCLUSIONS

Overall, declining soil water availability and plant leaf habit impacted the bacterial community. However, stronger effects of deciduous and evergreen tree species on bacterial community and N‐cycling function than short‐term drought had been proved in this study. Deciduous and evergreen tree species showed different plant traits in carbon fixation and growth. As a linkage between above and belowground, root biomass played key roles in affecting bacterial community structure and soil N cycling. However, our research is constrained by low sample size of deciduous and evergreen tree species. Further research on N‐cycling microbes affected by plant functions and plant–soil interactions is required to predict the respective consequences of climate change.

## AUTHOR CONTRIBUTIONS

**Jiantong Liu:** Formal analysis (equal); visualization (equal); writing – original draft (equal). **Xinyu Wang:** Data curation (equal); software (equal); writing – original draft (equal). **Lin Liu:** Data curation (equal); software (equal); visualization (equal). **Xuefeng Wu:** Visualization (equal). **Zhichao Xia:** Funding acquisition (supporting); writing – review and editing (equal). **Qingxue Guo:** Data curation (equal); funding acquisition (lead); methodology (lead); project administration (lead); writing – original draft (equal).

## CONFLICT OF INTEREST

The authors declare that they have no conflict of interest.

## Supporting information


Appendix S1.

## Data Availability

All bacterial data are available at the National Center for Biotechnology Information under BioProject ID PRJNA821928. Data of soil properties and plant biomass were input in Dryad. https://doi.org/10.5061/dryad.2547d7wqs.
