# Supplementary material for: Rhizosphere soil bacterial communities and nitrogen cycling affected by deciduous and evergreen tree species
Source: Ecol Evol. 2022 Jul 13;12(7):e9103. doi: 10.1002/ece3.9103 (PMC9277416; doi:10.1002/ece3.9103)
Supplement: Supplementary file 1 — Appendix S1. [file ECE3-12-e9103-s001.docx]

**Supplementary materials**

***Processing of sequencing data***

The raw 16S rRNA gene sequencing reads were demultiplexed, quality-filtered by fastp version 0.20.0 and merged by FLASH version 1.2.7 with the following criteria: (i) the 300 bp reads were truncated at any site receiving an average quality score of <20 over a 50 bp sliding window, and the truncated reads shorter than 50 bp were discarded, reads containing ambiguous characters were also discarded; (ii) only overlapping sequences longer than 10 bp were assembled according to their overlapped sequence. The maximum mismatch ratio of overlap region is 0.2. Reads that could not be assembled were discarded; (iii) Samples were distinguished according to the barcode and primers, and the sequence direction was adjusted, exact barcode matching, 2 nucleotide mismatch in primer matching.

Operational taxonomic units (OTUs) with 97% similarity cutoff were clustered using UPARSE version 7.1, and chimeric sequences were identified and removed. The taxonomy of each OTU representative sequence was analyzed by RDP Classifier version 2.2 against the 16S rRNA database using confidence threshold of 0.7.

***Real-time quantitative PCR (qPCR) for N-cycling genes***

The DNA extractions for 16S rRNA gene sequencing were also used for the qPCR of targeted AOA, AOB, *nifH*, *nirS* and *nirK* genes. Each PCR was conducted in 20 μl total volume that contained 10 μl ChamQ SYBR Color qPCR Master Mix (Nanjing, China), 6.4 μl H2O, 0.8 μl 5 μM forward primer, 0.8 μl 5 μM reverse primer and 2 μl DNA using ABI 7300 (Applied Biosystems, USA). For all these functional genes, an initial denaturation step was 95 ^o^C for 5 min, followed by forty cycles of 95 ^o^C for 5 s. The annealing temperatures were as follows: 58 ^o^C (AOA), 60 ^o^C (*nifH* and *nirK*) or 55 ^o^C (*nirS* and AOB). The final extension temperature was 72 ^o^C for 40 s. Serial dilutions of plasmids derived from cloned targets (10^-2^-10^-8^) were used to generate standard curves per functional gene.

**Table S1** Rhizosphere soil’s chemical properties of four plant species at two soil water levels.

|  |  | pH | SOM (g/kg) | TP (g/kg) | AP (mg/kg) | TN (g/kg) | NH_4_^+^-N (mg/kg) |
| --- | --- | --- | --- | --- | --- | --- | --- |
| *Liquidambar formosana* | Control | 7.52±0.08 | 13.70±0.71 | 0.86±0.05 | 51.11±1.42 | 0.77±0.03 | 4.49±0.33 |
|  | Drought | 7.46±0.05 | 11.92±0.41 | 0.88±0.04 | 54.42±1.82 | 0.75±0.03 | 3.70±0.20 |
| *Alnus cremastogyne* | Control | 7.73±0.05 | 13.27±0.59 | 0.85±0.07 | 48.34±6.50 | 0.72±0.04 | 2.73±0.18 |
|  | Drought | 7.82±0.05 | 12.26±0.49 | 0.85±0.03 | 43.36±7.02 | 0.69±0.02 | 1.44±0.07 |
| *Cunninghamia lanceolata* | Control | 7.56±0.07 | 13.56±0.53 | 0.83±0.03 | 39.07±1.75 | 0.80±0.05 | 4.17±0.12 |
|  | Drought | 7.64±0.05 | 12.11±0.35 | 0.75±0.01 | 42.94±2.13 | 0.68±0.01 | 3.12±0.25 |
| *Pinus massoniana* | Control | 7.41±0.02 | 14.67±0.71 | 1.02±0.05 | 73.32±2.80 | 0.88±0.02 | 3.59±0.27 |
|  | Drought | 7.47±0.04 | 12.40±0.37 | 0.82±0.03 | 63.14±2.42 | 0.71±0.04 | 2.94±0.14 |
| *P* value |  |  |  |  |  |  |  |
| Leaf habit |  | NS | NS | NS | NS | NS | NS |
| Drought |  | NS | *** | NS | NS | *** | *** |
| Leaf habit × Drought |  | NS | NS | NS | NS | ** | NS |

SOM: soil organic matter, TP: total phosphorus, AP: available phosphorus, TN: total nitrogen, NH_4_^+^-N: ammonium. Two-way ANOVA was used to check plant leaf habit, drought and their interactions on soil chemical properties. Different lower-case letters in column indicate significant differences. Significant effects of factors and interactions (×) are indicated. NS – not significant, ***: *P* ≤ 0.001, **: 0.01 *≤ P* < 0.001.





**Figure S1** The net photosynthetic rate of deciduous and evergreen plants at two soil water levels. Two-way ANOVA was used to check plant leaf habit, drought and their interactions on the net photosynthetic rate. *P* values were shown. D: drought effect; L: plant leaf habit effect; D*L: the interactions between drought and plant leaf habit. Post hoc tests were conducted to discover differences among treatments with Tukey’s *b* tests. Different letters indicated significant differences.


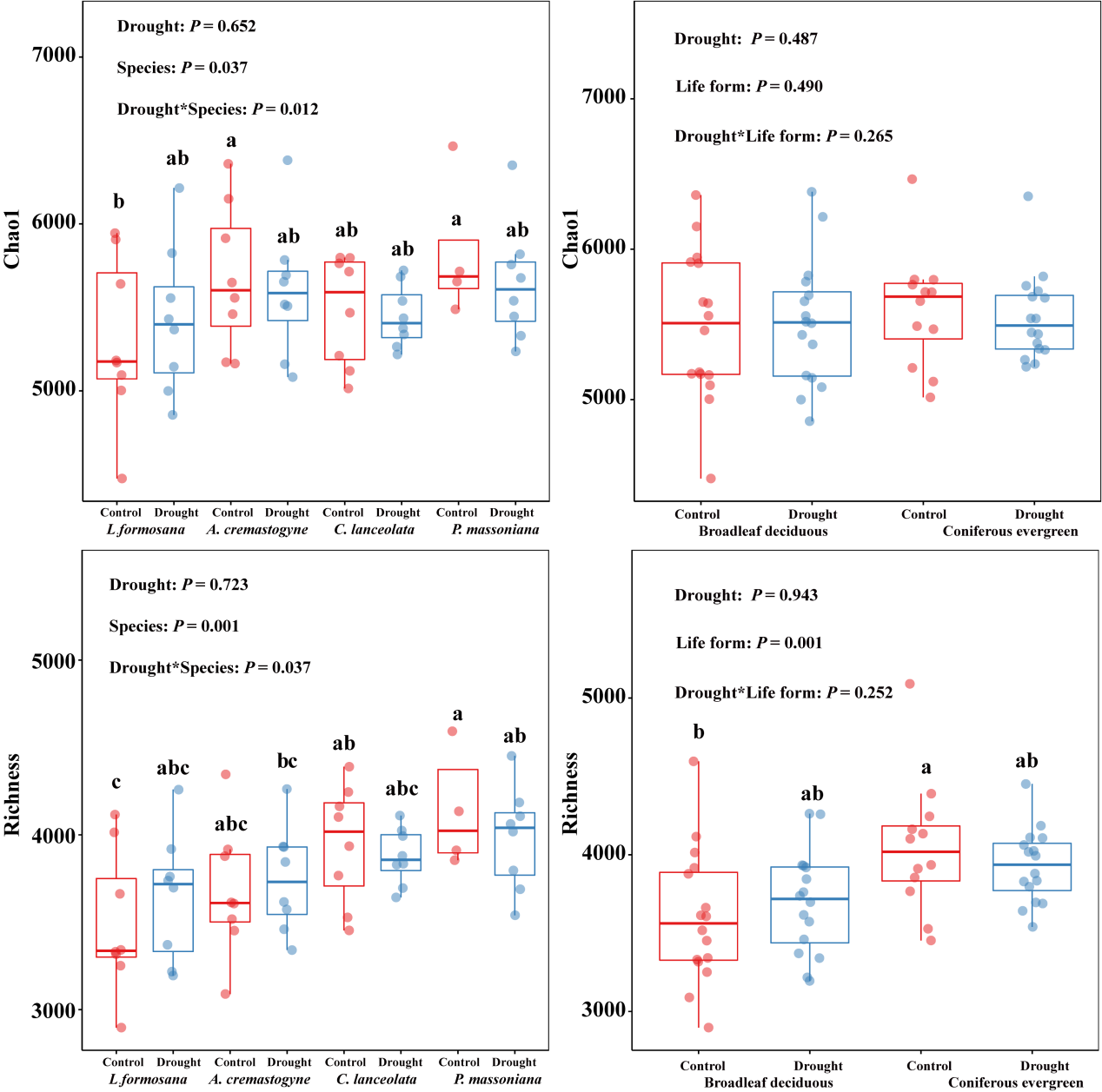


**Figure S2** Bacterial index of Chao 1 and richness in the soil of two deciduous (*Alnus cremastogyne* and *Liquidambar formosana*) and two evergreen (*Cunninghamia lanceolata* and *Pinus massoniana*) tree species in response to two water regimes. A two-way ANOVA was used. Post hoc tests were used to test differences among treatments with Tukey’s *b* tests. Different letters indicate significant differences.


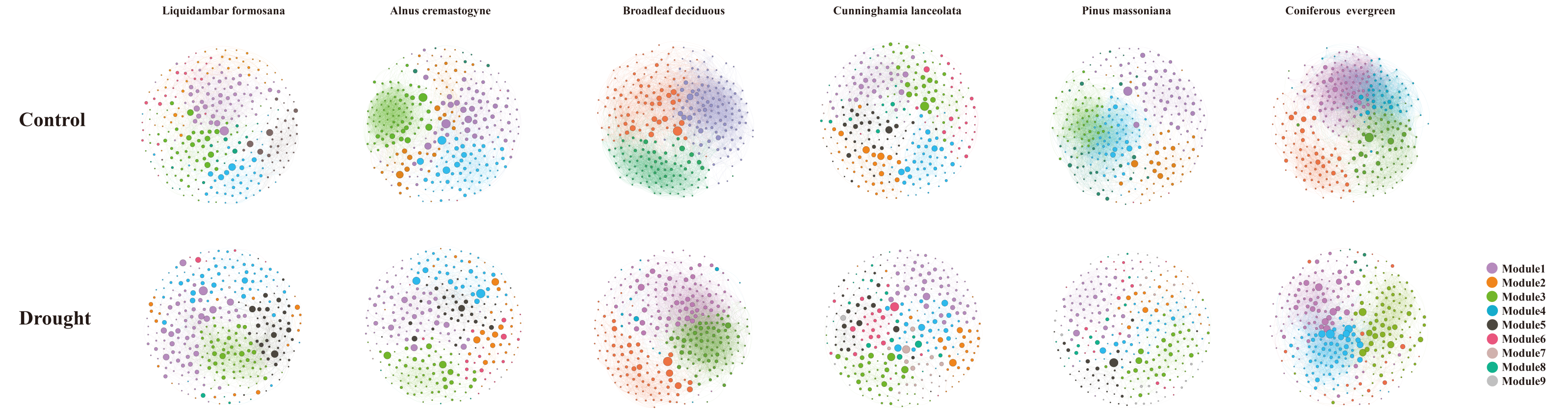


**Figure S3** The module of bacterial co-occurrence network in responding to two soil water levels. *Cunninghamia lanceolata* and *Pinus massoniana* belong to the evergreen species, *Alnus cremastogyne* and *Liquidambar formosana* belong to the deciduous species.


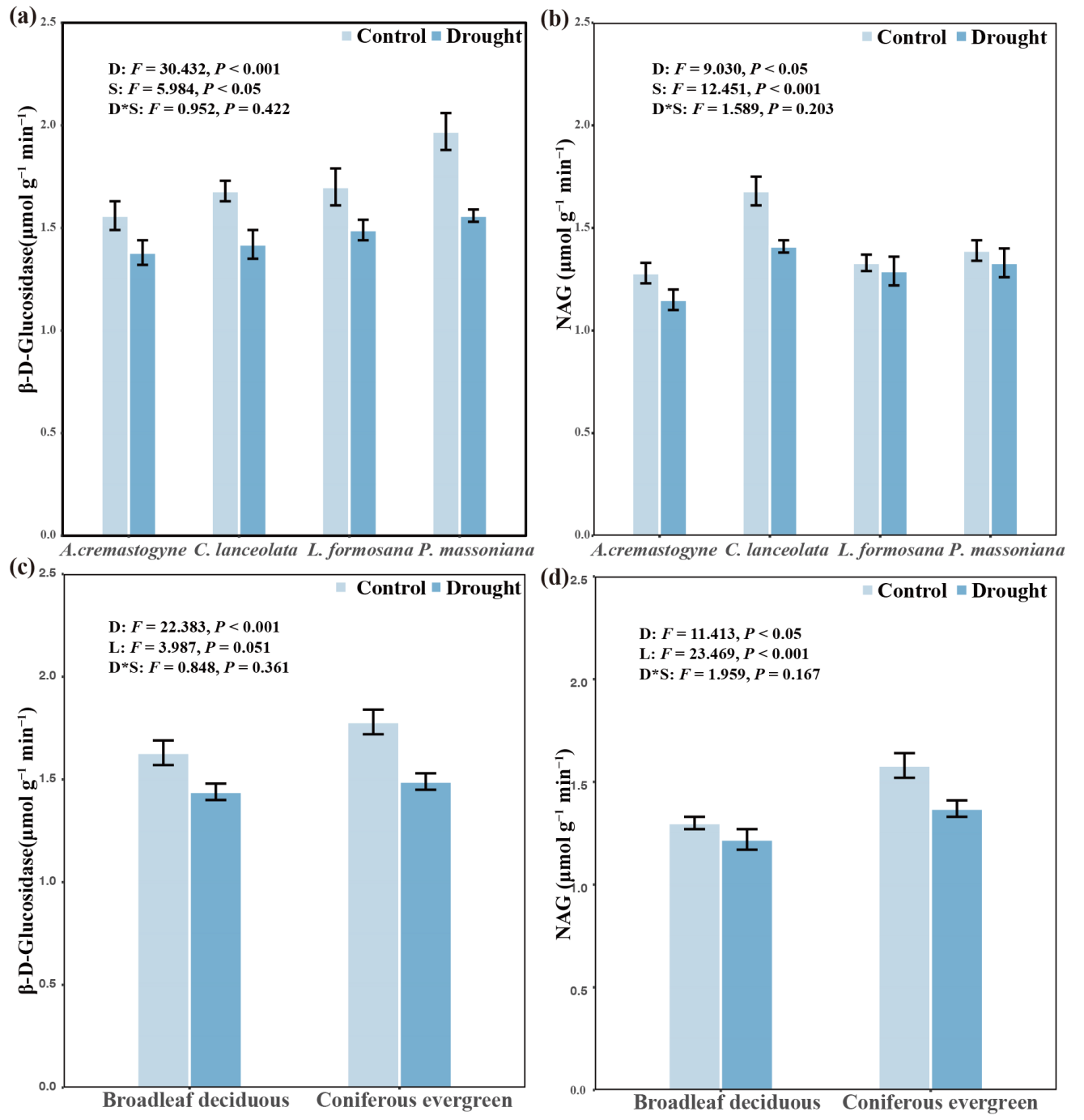


**Figure S4** The β-1,4-N-acetylglucosaminidase (NAG) and β-D-glucosidase activity of rhizosphere soil of each species and plant leaf habit. Two-way ANOVA was used to check plant leaf habit, drought and their interactions. *P* values were shown. D: drought effect; L: plant leaf habit effect; D*L: the interactions between drought and plant leaf habit.


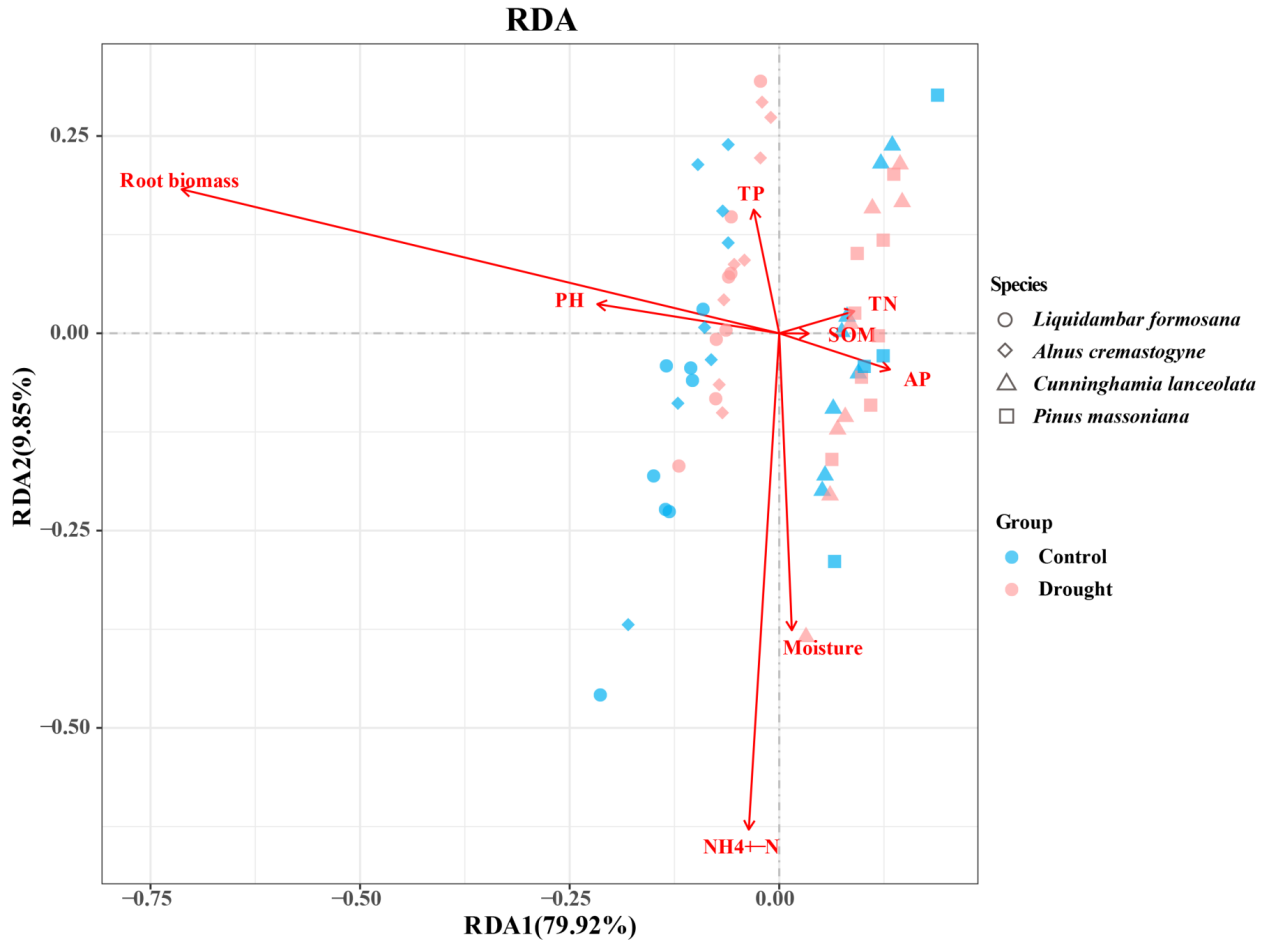


**Figure S5** Distance-based redundancy analysis (db-RDA) to assess effects of root biomass and soil properties on bacterial communities. SOM: soil organic matter, TP: total phosphorus, AP: available phosphorus, TN: total nitrogen, NH_4_^+^-N: ammonium, Moisture: soil water content.


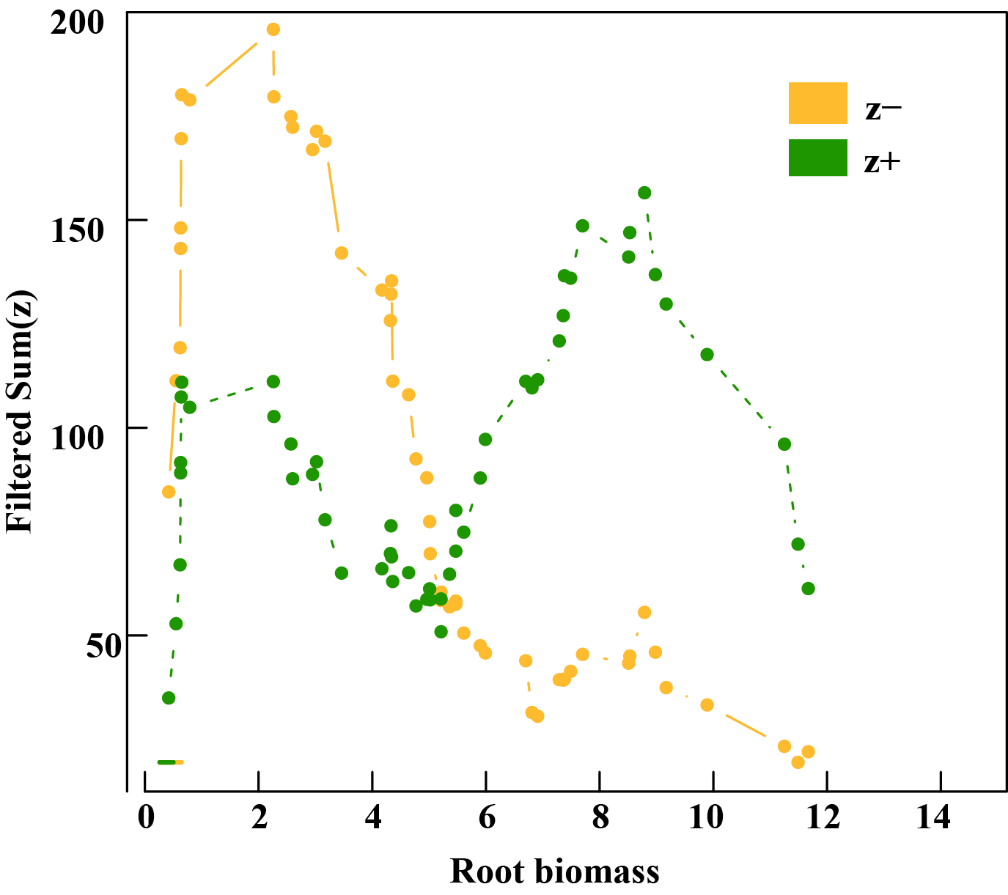


**Figure S6** Threshold Indicator Taxa Analysis (TITAN) sum (z-) and sum (z+) values corresponding to all candidate change points along the root biomass gradient. Yellow and green vertical lines represent the cumulative frequency distribution of change points (thresholds) among 500 bootstrap replicates for sum (z-) and sum (z+), respectively.

**
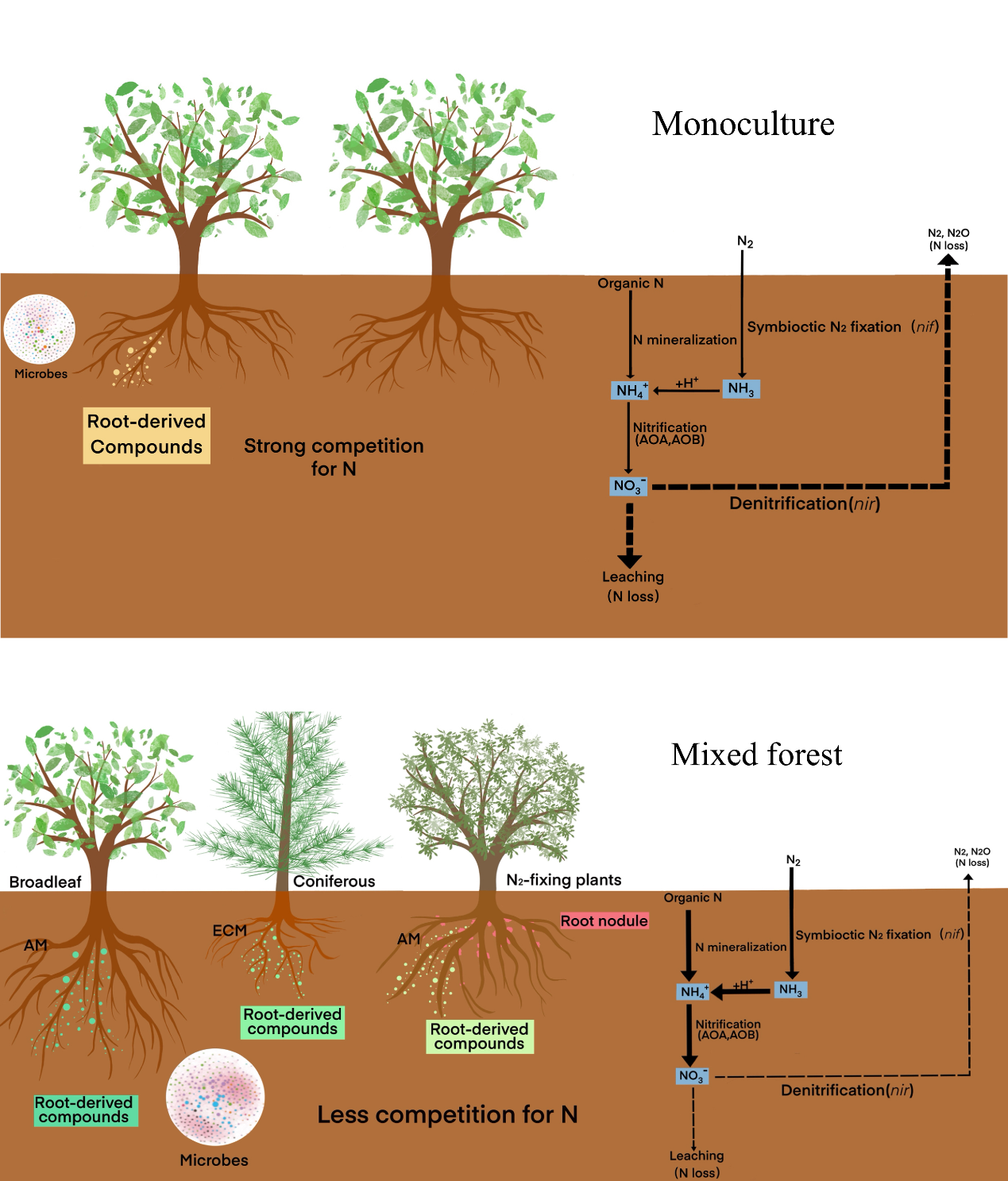
**

**Fig. S7** A simple framework to explain different impacts of monoculture and mixed forest on soil N cycle and drought resistance. Different color of circles represent root exudations of different plant leaf habit. Pink dots at the roots of the N_2_-fixing plant represent root nodules. Plants in monocultures show strong N competition due to the identical preference of N forms between plants (NH_4_^+^ or NO_3_^-^). More bacteria and fungi can be recruited by diverse plant communities and more stable and connected microbial community increases plant resistance to drought. Mixed forests greatly increase soil N availability through promoting N input, increasing soil N retention. N loss through leaching and denitrification is reduced in mixed forests because NO_3_^-^ can be more efficiently captured and used by different plant species. Higher N demand of deciduous plants accelerates N cycling, from which evergreen plants may benefit in a mixed forest. Symbiotic mycorrhiza contribute to niche separation of different plants suggesting less competition for N and higher forest productivity
